# nFeS Embedded into Cryogels for High-Efficiency Removal of Cr(VI): From Mechanism to for Treatment of Industrial Wastewater

**DOI:** 10.3390/gels10010056

**Published:** 2024-01-11

**Authors:** Peng Xu, Shaojun Jiang

**Affiliations:** 1 School of Environment, South China Normal University, Guangzhou 510006, China; xupeng@gdim.cn; 2 Institute of Agricultural Resources and Environment, Guangdong Academy of Agricultural Sciences, Guangzhou 510640, China

**Keywords:** metal pollution, adsorption, reduction, poly sodium acrylate, industrial wastewater

## Abstract

Most studies have focused on complex strategies for materials preparation instead of industrial wastewater treatment due to emergency treatment requirements for metal pollution. This study evaluated sodium polyacrylate (PSA) as a carbon skeleton and FeS as a functional material to synthesize PSA-nFeS material. The characteristics and interactions of PSA-nFeS composites treated with hexavalent chromium were analyzed by means of various techniques, such as scanning electron microscopy-energy dispersive X-ray spectrometry (SEM-EDS), X-ray photoelectron spectroscopy (XPS), Fourier transform infrared spectrometry (FTIR), and atomic absorption spectroscopy (AAS). Adsorption-coupled reduction was observed to be the predominant mechanism of Cr(VI) removal. The feasibility of PSA-nFeS composites in reducing toxicity and removing of Cr(VI) from real effluents was investigated through column studies and material properties evaluation. The continuous column studies were conducted using tannery effluents to optimize feed flow rates, initial feed Cr(VI) concentration, and column bed height. The results revealed that PSA-nFeS composites are ideal for filling materials in portable filtration devices due to their lightweight and compact size.

## 1. Introduction

The widespread use of Cr and its compounds as raw materials for stainless steel [1], metal electroplating [2], leather industries [3], catalysis production [4], and pigment processing [5] and other industries has led to an increasing amount of Cr-containing wastewater being discharged into the environment, causing increasingly serious problems of Cr(VI) pollution in the water environment [6]. Compared to Cr(III), Cr(VI) is mobile and soluble, highly toxic [7] and can cause a variety of diseases.

In the past, a large number of chromium removal techniques have been applied in environmental remediation techniques, such as coagulation, precipitation, adsorption, reduction, ion exchange, electrodialysis and filtration, which can be applied individually or in combination [8]. There has been a focus on the reduction of Cr(VI) to Cr(III) and precipitation (coagulation). Some reducing agents such as NaBH_4_, FeSO_4_, Na_2_S_2_O_5_, NaHSO_3_, Na_2_S_2_O_3_, Na_2_SO_3_, sulfur-functionalized materials Fe-based materials and nZVI were added to pledge the reduction of Cr(VI) to Cr(III) [9,10]. In recent years, there has been a focus on the reduction of Cr(VI) to Cr(III); researchers have turned their attention to nano-FeS because of its large specific surface area, strong reducing ability, and high reactivity. However, due to the small particle size of nano-FeS, it is easy to agglomerate and oxidize [11,12,13]. This limits its ability to handle heavy metal ions. To overcome these drawbacks, researchers have developed many porous materials (e.g., zeolite, montmorillonite, carbon nanotubes, cryogel, etc.) to support it to enhance its dispersibility and reaction activity [8,14,15,16]. Among these materials, cryogel is widely used to support n-FeS due to its excellent stability, low cost, and abundant pore structure, which could immobilize nZVI particles and be convenient to be separated from reaction medium as well as retain their reactivity [15]. Furthermore, a novel monolithic composite (PSA-nFeS) has been reported by Ma [16]. It had been found that PSA-nFeS exhibited high removal of hexavalent chromium at pH 2–10, from 16.40% (41.30 mg/g) to 90.51% (282.43 mg) with an increase of nFeS. Similar, Jia et al. (2018) reported that PSA-nZVI composites by incorporating nanoscale zero-valent iron particles (nZVI) within poly (sodium acrylate) (PSA) cryogels, via ion exchange and subsequent in-situ reduction. And PSA-nZVI composites demonstrated significantly enhanced efficiency in eliminating Cr(VI) and total Cr, particularly in scenarios of high removal capacity and a wide pH application spectrum (pH 4–10). Unfortunately, the results of these studies are at the stage of batch experiments, and it is not clear whether they fail in practical water treatment engineering [15,16]. The implementation of integrating chromate removal methods into the actual water matrix presents a complex challenge. Wastewater contaminated with Cr(VI) comprises a multifaceted matrix with not just Cr(VI) but also substantial amounts of solids, chemical oxygen demand (COD), various heavy metal ions with positive and negative charges, ambient electrolytes, organic pollutants, and oil/grease [8,17,18,19].

Previous studies have shown that the presence of oxygen, ion concentration, natural organic matter, and coexisting cations in the actual water environment affects the removal of Cr(VI) by FeS [16,17]. Due to the highly reducing nature of FeS, it is easily oxidized in the environment, and the removal performance of the composite material under adequate oxygen or anaerobic conditions needs to be further verified [20]. Dissolved organic matter (DOM) in the aqueous environment can adsorb on Fe-based materials and occupy reaction sites [15,19]. In addition, FeS has a strong binding ability with metal cations or forms metal sulfide precipitates [11]; moreover, their presence may occupy the reaction sites of FeS and compete with Cr(VI) for S(-II), while their concentrations also affect the removal process of Cr(VI) [21]. Loo et al. [22] explored the environmental effect of water treatment contingency with PSA crystalline gel-loaded metal particles. However, recycling and dynamic experiments can better simulate the actual water treatment situation, providing more information to reflect the actual performance of the material than static batch experiments. Thus, it is necessary to process the effluent and eliminate Cr(VI) prior to its release into the environment.

The study employed PSA-nFeS composites to eliminate Cr(VI) from both artificial and actual effluents. Assessing the performance of PSA-nFeS composites involves examining the real impact of Cr(VI) elimination by PSA-nFeS, considering the various factors previously discussed. The research concentrated on identifying the key factors and potential methods for removing Cr(VI). Furthermore, a series of dynamic and recycling studies were carried out to establish both theoretical and practical foundations for applying these materials in the context of environmental sustainability.

## 2. Results and Discussion

### 2.1. PSA-nFeS Composites Characterization

**Figure 1:**
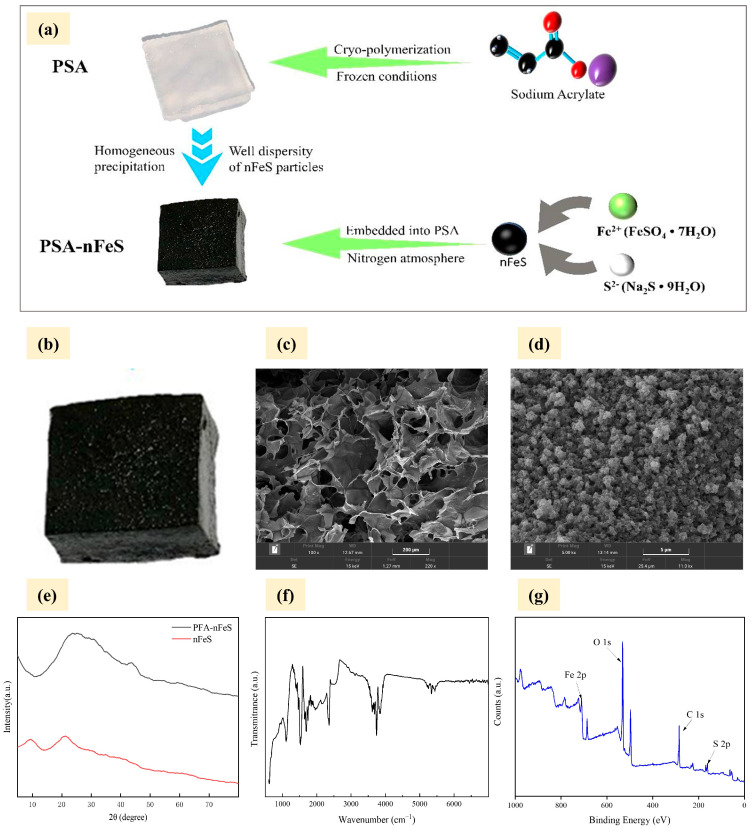
Synthesis steps of PSA-nFeS (**a**), digital photos (**b**), SEM images (**c**,**d**) of the PSA-nFeS composites; XRD (**e**), FTIR (**f**), and XPS (**g**) spectra of PSA-nFeS composites.

Acrylic ion exchange fiber PSA was synthesized using the co-polymerization technique, with SA, APS, MBAm and TEMED. In brief, the synthesis of PSA-nFeS composites consists of two steps: first, the synthesis of PSA condensation gel is carried out by the ion exchange process, followed by uniform precipitation of nFeS on PSA condensation gel (Figure 1a). Figure 1a illustrates the SEM visuals of the created PSA-nFeS composite materials. It was revealed that the PSA cryogel possessed a highly porous, open, and interconnected structure with a sleek surface. Figure 1b,c show that the surface of the PSA cryogel is uniformly loaded with black material. According to previous studies, it was proven that it was nFeS loaded on the gel surface [16]. The XRD patterns of the synthesized FeS nanoparticles, initial and final PSA-nFeS composites are shown in Figure 1d. The distinct peaks noted were the planes corresponding to the FeS and Na_2_SO_4_ peaks, which had similar results in previous studies [23]. The FTIR spectra data of the synthesized PSA-nFeS composites are shown in Figure 1e. The spectrum peak of PSA cryogel appeared approximately at 3259 cm^−1^ (O/N–H bands) and 2945 cm^−1^ (C–H bands), which indicated the role of the PSA cryogel as the basic skeleton. The PSA-nFeS composites were comparatively studied with XPS to explain the change in its surface further. The XPS spectra of C 1s, O 1s, S 2p and Fe 2p are shown in Figure 1f. Peaks of S 2p and Fe 2p were clearly observed by XPS demonstrating that FeS was successfully attached to PSA, and this phenomenon was also confirmed by EDS analysis (Figure S2). The results implied that nFeS was successfully loaded on the PSA cryogel. The mechanism of action was described in detail in our previous studies [16]. The results indicated that reduction of Cr(VI) might contribute the majority of its removal while adsorption played a minor role, in which Fe(II) and S(-II) acted as reductants.

**Figure 2:**
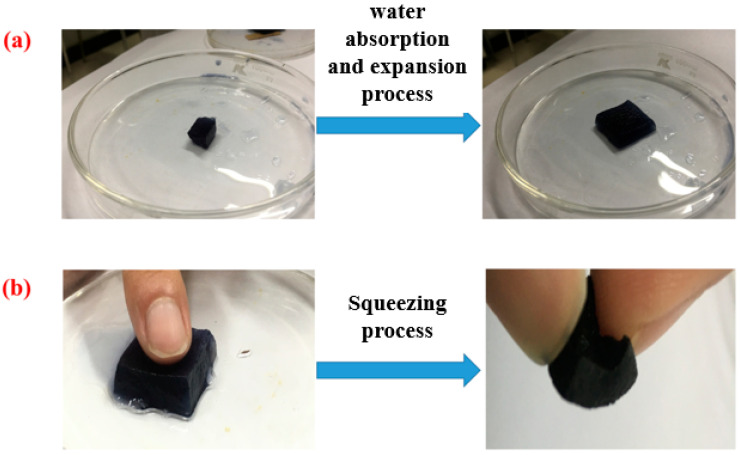
(**a**) PSA-nFeS swelling comparison and (**b**) effect after squeezing.

Furthermore, in this study, the emergency treatment effect of PSA-nFeS composites was evaluated by extrusion experiments (Figure 2). The results show that the elapsed time for water absorption and expansion of PSA-nFeS composites was found to be within 10 s (Table 1). Comparing the results of previous studies, the PSA-nFeS composites are more advantageous for the removal of Cr (Table S2). In addition, Figure 2b shows that the volume of PSA-nFeS material expands rapidly after water absorption. After a simple squeezing operation, the PSA-nFeS material regains its original volume size without water absorption, which has the advantages of short-time water absorption and expansion, significant volume change due to physical properties, high water absorption and desorption rate.

### 2.2. Effect of Environmental Factors on Cr Removal

**Figure 3:**
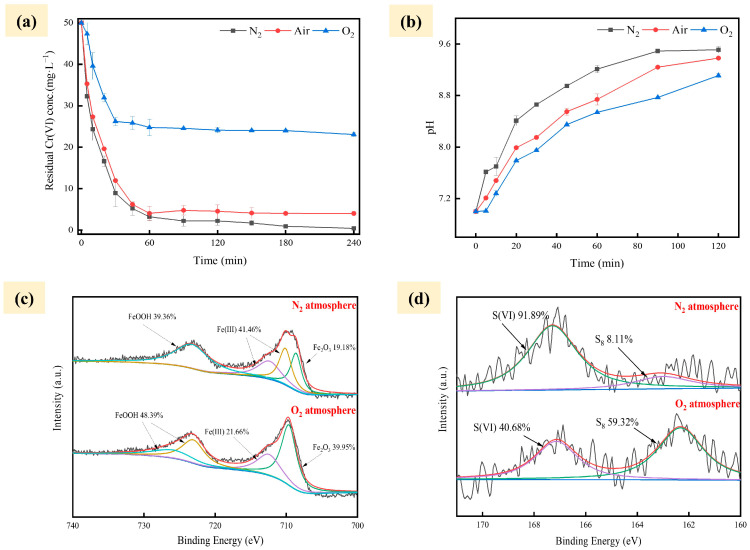
(**a**) Cr(VI) removal and (**b**) pH after reaction under different atmosphere, XPS spectra of Fe (**c**)/S (**d**) in reaction condition under different atmosphere.

Figure 3 shows the effect of PSA-nFeS material on the removal of Cr(VI) under different environmental factor changes. The results revealed that oxygen reduces the Cr(VI) removal rate from 90.51% to 51.90%. Cr(VI) removal rate reached 100% after excluding the oxygen interference. The presence of oxygen may disrupt the Fe-S bond within nFeS and thus affect the removal of Cr(VI) [25]. Sapsford et al. [26] found that oxygen can oxidize Fe(II) to form FeOOH, but its removal performance is significantly lower than that of FeS. The change in solution pH for each gas atmosphere in the reaction is shown in Figure 3b. The results indicated that the presence of oxygen in the reaction system has the negative effect of lowering the pH in the solution [1]. In particular, the oxidation of Fe^2+^ in FeS leads to the formation of H^+^, which leads to a decrease in the solution pH, as shown in Equation (1).

(1) $$O_{2}\left(aq\right)+4{Fe}^{2+}+6H_{2}O\to 4FeOOH\left(s\right)+8H^{+}.$$

XPS analysis was performed after the reaction to investigate the surface changes in PSA-nFeS material. The results showed that the main reaction product of PSA-nFeS under a nitrogen atmosphere is the Fe(III) type component (Figure 3a), which may be related to Cr-Fe complexation precipitation, while the main product under an oxygen atmosphere was FeOOH, and the reaction process corresponds to Equation (1). Moreover, Fe 2p spectra in Figure 3c illustrate that oxygen increases the loss of Fe^2+^ from the material surface. The results revealed that FeOOH was converted in the aqueous phase and adsorbed to the composite surface, which occupies the reactive site of the material and leads to the decrease in the material’s Cr(VI) removal efficiency. The main reaction product was SO_4_^2−^ under a nitrogen atmosphere, while the main product was S under an oxygen atmosphere. The results suggest that the removal of Cr(VI) is better when S is oxidized to higher valence elements [27].

**Figure 4:**
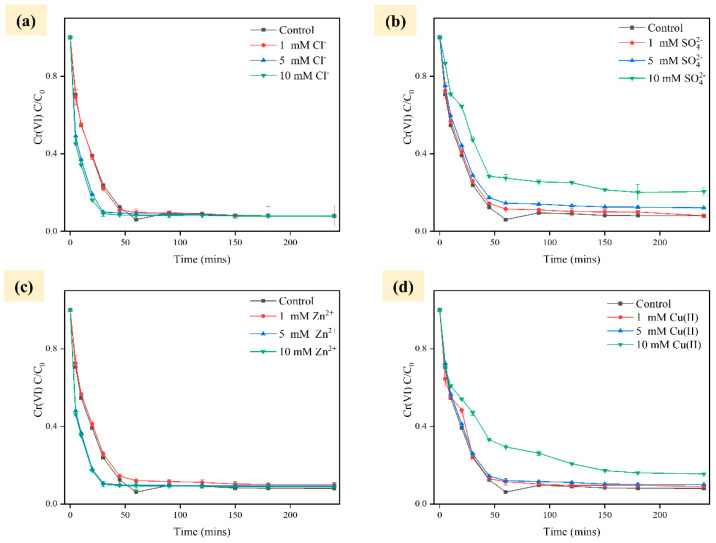
Elimination of Cr(VI) using PSA-nFeS under different ionic concentrations ((**a**) Cl^−^, (**b**) SO_4_^2−^, (**c**) Zn^2+^, (**d**) Cu^2+^).

Metal ions, anions, and dissolved organic matter are common constituents of wastewater. The presence of Zn^2+^and Cl^−^ (1 mM, 5 mM and 10 mM, respectively) in PSA-nFeS material significantly impacted the reduction of Cr(VI), indicating its resilience against the adverse effects of these ions, as illustrated in Figure 4. The results indicated that Fe^2+^ enhanced Cr removal due to the supplementation of more iron species to precipitate in the iron redox process due to the dissolution rate of FeS increasing with the increase in electrolyte ion concentration. Thus, dissolving more Fe and S to react with Cr(VI) is advised [28]. Moreover, Cl^−^ at higher concentrations accelerates the precipitation of iron oxides to promote reactive sites, while slowing down the passivation of iron on the material surface [29]. However, ambient Zn^2+^ and Cl^−^ did not interfere or inhibit the actual effect of Cr(VI) removal by PSA-nFeS composites. The results indicated that the effect of different SO_4_^2−^ concentrations on the reaction system was variable. The removal of Cr(VI) from the system remained unaffected at low SO_4_^2−^ concentrations. At a concentration of 10 mM, the removal of Cr(VI) by PSA-nFeS showed a slight decrease. The adsorption of SO_4_^2−^ onto the oxide may lead to the formation of complexes with iron on the material surface, thereby maintaining or accelerating iron dissolution [30]. It has also been suggested that sulfates act as electron acceptors for dissolution. Surface passivation may result from its reaction with FeS surfaces [31]. Additionally, the coverage of active sites on the material surface by SO_4_^2−^ can cause a reduction in the number of reactive sites [32]. As the Cu^2+^ rapidly increased, the Cr(VI) removal rate significantly slowed down. Zhao et al. [33] found that Cu^2+^ potential reduction was higher than that of Fe^2+^, leading to zero valence on the PSA-nFeS material surface and forming a primary cell around FeS particles, promoting electron transfer. The findings showed that Cu^2+^ wrapping diminishes material reaction sites, reducing Cr(VI) removal at 10 mM Cu^2+^.

The effect of DOM on the oxidation of Cr removal was investigated by using HA as the model compound. The results showed that HA was effective in the concentration range of 1–10 mg/L for the removal of Cr(VI) from PSA-nFeS (Figure S3). The presence of functional groups like quinones on HA with high electron shuttle efficiency accelerated the reduction of Cr(VI) removal by the material [34].

### 2.3. Columnar Experiment

**Figure 5:**
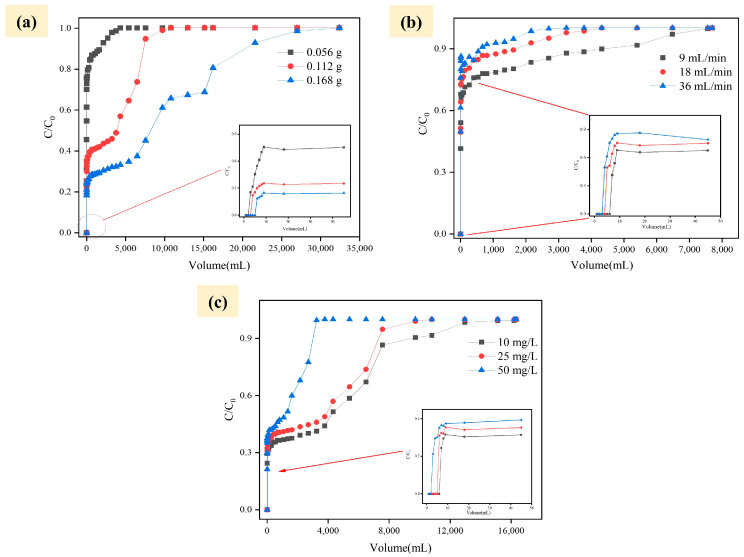
Removal of Cr(VI) ration using PSA-nFeS under different process parameters. (**a**) dosage of PSA-nFeS; (**b**) speed of reaction system; (**c**) original Cr(VI) concentration.

Combined with the dynamic columnar experiment, the effect of different parameters (concentration, filling volume, flow rate) on removing Cr in the actual wastewater effect was investigated. The experiments were carried out at a flow rate of 1.08 L/h and an initial concentration of 25 mg/L of Cr(VI) to evaluate the effect of PSA-nFeS with different filler amounts on the removal of Cr(VI). Figure 5a demonstrates that an increase in filling volume from 0.056 g to 0.168 g leads to a significant increase in feed volume (4500 mL to 32,000 mL). The result indicates that Cr(VI) removal increases with the increase in the filling mass. As the material’s filling mass increases, the mass transfer zone and depth also increase, increasing available reaction sites and reducing substances, thereby enhancing removal capacity and penetration site volume. The study found that a decrease (1.08 L/h to 0.54 L/h) in influent flow rate led to an increase in effective treated water volume (4.50 L to 7.80 L) and improved removal efficiency of Cr. However, an increase in the influent flow rate to 1.62 L/h resulted in a high effluent Cr(VI) concentration due to the large influent flow rate and short contact time between the material and pollutants. Model fitting of the curves for the influent flow rates of 9, 18, and 36 mL/min resulted in the removal of Cr(VI) by PSA-nFeS material, which was 233.71, 214.49, and 195.71 mg Cr(VI)/g nFeS, respectively (Figure 5b). The column experiment showed an increase in flow rate, suggesting that the water capacity of PSA-nFeS material could be reduced to some extent.

Figure 5c demonstrates that the experimental column penetrated at a concentration of 50 mg/L around 3800 mL of feed solution, with penetration effects of 25 mg/L and 10 mg/L for 10,000 mL and 15,000 mL of feed solution, respectively. The increased driving force at the interface between the sorbent–sorbate may be due to the increased concentration gradient due to the rise in Cr(VI) ions [35,36]. The experiment demonstrates that the expansion of the PSA-nFeS material due to liquid absorption increases its resistance to the Cr(VI) solution. The penetration curve increases with the initial concentration of Cr(VI) solution and the volume amount of the penetration point [37,38]. The lower the initial wastewater concentration, the better the contact reaction between PSA-nFeS and Cr(VI), leading to higher removal rates of Cr(VI). In practical applications, the lower the concentration of Cr(VI)-contaminated groundwater, the more effective the reaction is when passing through a filled column containing PSA-nFeS.

In addition, the removal of co-existing substances from wastewater by the reaction system was also determined in this study. From the results in Table S1, it can be tentatively concluded that the reduction and adsorption efficacy of the PSA-nFeS composites is also effective in reducing the concentration of other co-existing ions, but with a loss of some of the reduction properties. So, pre-treatment may be considered when performing actual industrial wastewater treatment.

### 2.4. Model Analysis

The study reveals that the KTh in the Thomas model increases with an increase in flow rate but decreases with an increase in the filling volume. The rate constant KTh is influenced by the change in filling volume, possibly due to an increase in mass transfer resistance and required diffusion power. Thus, the greater the height, the higher the resistance, and the response rate reduces as the filling quantity progressively rises. The removal capacity of PSA-nFeS material decreases with increasing KTh, suggesting that the material’s driving force for Cr(VI) removal is the difference between the Cr(VI) concentration on its surface and the wastewater concentration. Furthermore, the data (Table 2) show that the Yoon–Nelson model’s fitted correlations have a stronger R^2^ than the Thomas model’s, indicating that the Yoon–Nelson model can more accurately express. The Yoon–Nelson model’s R^2^ is higher than that of the Thomas model, indicating it better describes the removal rate and process of Cr(VI) by PSA-nFeS, and confirming that adsorption is not dominant in this dynamic process.

## 3. Conclusions

The present study investigated the potential of PSA-nFeS composites for Cr(VI) removal materials in emergency wastewater treatment. A range of analytical methods were used to evaluate the biosorbent, including SEM, FTIR, XPS, XRD, and AAS. The mechanism of elimination was discovered to be adsorption-coupled reduction. The inhibitory effect of oxygen on the elimination of Cr(VI) by PSA-nFeS composites was observed during the oxidation of Fe and S. The removal efficacy of FeOOH and S8 was found to be lower in comparison to that of FeS. The effect of ions and humic acid coexisting was negligible. Dynamic Cr(VI) removal improved with increasing filling volume, input water concentration, and flow rate. The Yoon–Nelson model provided a description of the dynamic removal process. The potential application of PSA-nFeS composites in emergency treatment scenarios for the detoxification of industrial effluents containing Cr(VI) can be attributed to their mobility and lightweight nature.

## 4. Materials and Methods

### 4.1. Materials and Reagents

The PSA-nFeS synthesis includes a variety of raw materials, such as Ammonium persulfate (APS, 98%), Sodium acrylate (SA, 98%), N, N′-methylenebis (MBAm, 98%), N, N, N′, N′-tetramethylethylenediamine (TEMED, 99%), FeSO_4_·7H_2_O, (Na_2_S·9H_2_O), K_2_Cr_2_O_7_, (99.9%),1,10-phenanthroline and 1, 5-diphenylcarbazide (DFC, 98%), which were acquired from the chemical reagent manufacturers Macklin (Shanghai, China) and Tianjin Damao chemical reagent factory companies (Tianjin, China). Actual industrial wastewater was collected from a Tanning workshop of a factory (Guangdong, China) in Guangdong Province. The physicochemical parameters of wastewater are given in Table S1. The study utilized analytical grade reagents without further purification. The water used in the experimental procedure was deioni periment, except for the N_2_-purification requirement.

### 4.2. Preparation of PSA-nFeS and Reactor

The creation of PSA-nFeS adhered to the procedure outlined by our previous study [16]. The synthesized PSA-nFeS was washed using anaerobic water and then freeze-dried and set aside.

The column reactor (Figure S1), which was made of a plexiglass column, consisted of four parts: upper- and lower-end rubber plug, water transmission line, plexiglas post with a hairy mouth and peristaltic pump serves (Figure S1a). The column reactor length and inner diameter were 50 cm and 6 cm, respectively. In addition to the filling of the medium in the reaction, a layer of the screen was first placed on the bottom of the reactor, followed by 1 cm of quartz sand; then, the material of PSA-nFeS was filled according to the different filling doses, and finally the screen and quartz sand were added (Figure S1b).

### 4.3. Environmental Factors

The experiments investigated the effects of dissolved oxygen, interfering ions and organic matter for PSA-nFeS composite reaction with Cr(VI). To observe the effect of dissolved oxygen on Cr removal, two PSA-nFeS composites were added to 50 mL of a 50 mg/L Cr(VI) solution. The pH of the system was adjusted to 7.00 ± 0.02 with a 0.1 mol/L NaOH solution. Cr(VI) concentration was measured after 180 min of reaction. Aerobic and anaerobic conditions are regulated by the charging of O_2_ and N_2_ during the reaction. Other steps were the same as in the control group.

The coexisting cations of Zn^2+^ and Cu^2+^ and the coexisting anions of Cl^−^ and SO_4_^2−^ were selected to investigate the effect of different interfering ions on the removal of Cr. Three concentrations were set, 1 mM, 5 mM and 10 mM. First, different concentrations of interfering ions were mixed with the Cr solution and prepared in 50 mL of a 50 mg/L Cr(VI) solution, and then two PSA-nFeS composites were added at different time intervals (5 min, 10 min, 20 min, 30 min, 45 min, 60 min, 90 min, 120 min, 150 min, 180 min, 240 min, 150 min, 180 min, 240 min), and Cr(VI) concentrations were measured.

A 1000 mg/L standard solution of humic acid (HA) was prepared by weighing 1.00 g of HA, while 50 mL of a 50 mg/L Cr(VI) solution was prepared by transferring different concentrations of HA solution into a reaction flask. Two PSA-nFeS composites were added, and samples were taken after 180 min of reaction and measured.

### 4.4. Column Experiments

During the test, the columns were placed at room temperature. At the beginning of the reaction, the Cr(VI) solution was pumped from the bottom of the column. After the solution was infiltrated completely, the filtrate was collected once from the top of the column at regular intervals, passed through the membrane, and left to be measured. After the reaction, reaction media were collected at the sampling points in the middle part of the column and air-dried and characterization of reaction media. Each experiment was performed in triplicate at room temperature. The wastewater used in the reaction process was diluted by industrial wastewater.

### 4.5. Dynamic Experimental Model

The Thomas model and the Yoon–Nelson model were used to fit the experimental data, and their respective model equations are shown in Equations (2) and (3). The Thomas model is a conventional dynamic adsorption model, and the Yoon–Nelson model, which has no limitation on the characteristics of filler and reaction process, was added for comparison because the process of Cr(VI) removal by PSA-nFeS material is not a simple adsorption process [17,18].

(2) $$\mathrm{Thomas}\;\mathrm{model}:\;\frac{C_{t}}{C_{0}}=\frac{1}{1+exp\left[\frac{K_{T}}{Q}\left(q_{0}m-C_{0}V\right)\right]},$$

where C_t_ is the Cr(VI) concentration in the effluent at time t (mg/L); C_0_ is the influent Cr(VI) concentration (mg/L); K_T_ is the Thomas model rate constant (L^2^mg^−1^-h^−1^); q_0_ is the amount of pollutant removed at equilibrium (mg Cr(VI)/g nFeS); m is the total mass of nFeS in the filled composite in the experimental column (g); Q is the influent flow rate (L/h); V is the volume of solution passing through the experimental column (L); and t is the experimental column run time (h).

(3) $$\mathrm{Yoon}-\mathrm{Nelson}\;\mathrm{model}:\;q_{0}=\frac{C_{0}Qt_{2}-\int_{0}^{t_{2}}C_{e}Qdt}{m},$$

where $C_{e}$ is the Cr(VI) concentration in the effluent at *t*_2_ (mg/L); $C_{0}$ is the Cr(VI) concentration in the influent (mg/L); $q_{0}$ is the amount of pollutant removed at equilibrium (mg Cr(VI)/g nFeS); m is the total mass of nFeS in the filled composite in the experimental column (g); *Q* is the influent flow rate (L/h); *t*_2_ is the removal saturation time (h).

### 4.6. Analysis Methods

Flame atomic absorption spectroscopy (AAS, Hitachi ZA3000, Tokyo, Japan) was used to determine the Cr content in the solution. X-ray photoelectron spectroscopy (XPS, ESCALAB 250, Thermo Fisher, Waltham, MA, USA), X-ray powder diffraction test (XRD, Japanese Science Company, Rigaku, Tokyo, Japan), field emission scanning electron microscopy (SEM, Hitachi SU8020) and Fourier transform infrared spectrometry (FTIR, Bruker, VERTEX 70, Ettlingen, Germany) were used to measure material surface composition and chemical information.

## Figures and Tables

**Table 1:** Comparison of swelling times of different cryogel materials.

Materials	Time (s)	References
PSA-Ag	15	[24]
PSA-AgNP	10	[22]
PSA-nFeS	9	This study

**Table 2:** The parameters of dynamic experiment.

C_o_/ (mg/L)	Filling Doses/ (g nFeS)	Q/ (L/h)	Thomas Model			Yoon–Nelson Model	
			K_T_/(L/h·mg)	q_0_/(mg/g)	R^2^	q_0_/(mg/g)	R^2^
25	0.056	0.54	0.4884	256.5410	0.9137	233.7130	0.9381
25	0.056	1.08	0.5357	214.7280	0.9915	214.4920	0.9941
25	0.056	1.62	1.3206	206.0150	0.9318	195.7140	0.9567
25	0.056	1.08	0.5357	214.7280	0.9915	214.4920	0.9941
25	0.112	1.08	0.1921	230.2450	0.9508	218.7330	0.9762
25	0.168	1.08	0.1285	241.0290	0.9606	228.9770	0.9862
10	0.112	1.08	0.1923	235.7700	0.9529	228.6160	0.9783
25	0.112	1.08	0.1921	230.2450	0.9508	218.8690	0.9762
50	0.112	1.08	0.2763	170.3140	0.9412	161.7990	0.9456

## Data Availability

The data presented in this study are available on request from the corresponding author. The data are not publicly available due to the privacy of the patients who assisted in the research.

## Supplementary Materials

The following supporting information can be downloaded at: https://www.mdpi.com/article/10.3390/gels10010056/s1, Figure S1. Column experiments of reaction system. Figure S2. SEM-EDS elemental mapping images of PSA-nFeS. Figure S3. Effect of Cr(VI) removal capacity of HA. Table S1. Physico-chemical parameters of industrial effluents. Table S2. A comparison of treatment effect for Cr ions by different materials. References [15,16,39,40,41,42,43,44,45] are cited in the supplementary materials.

## References

[1] Li C., Zhang P., Zeng L., Yu L., Li D. (2023). Study on preparation of glass-ceramics from municipal solid waste incineration (MSWI) fly ash and chromium slag. J. Build. Eng..

[2] Dhal B., Thatoi H.N., Das N.N., Pandey B.D. (2013). Chemical and microbial remediation of hexavalent chromium from contaminated soil and mining/metallurgical solid waste: A review. J. Hazard. Mater..

[3] Sethuraman C., Srinivas K., Sekaran G. (2014). Pyrolysis coupled pulse oxygen incineration for disposal of hazardous chromium impregnated fine particulate solid waste generated from leather industry. J. Environ. Chem. Eng..

[4] Qasem N.A.A., Mohammed R.H., Lawal D.U. (2021). Removal of heavy metal ions from wastewater: A comprehensive and critical review. Npj Clean. Water.

[5] Lv D., Zhou J.S., Cao Z., Xu J., Liu Y.L., Li Y.Z., Yang K.L., Zimo Lou Z.M., Lou L.P., Xu X.H. (2019). Mechanism and influence factors of chromium(VI) removal by Sulfide-modified nanoscale zerovalent iron. Chemosphere.

[6] Hu Y., Peng X., Ai Z.H., Jia F.L., Zhang L.Z. (2019). Liquid nitrogen activation of zero-valent iron and its enhanced Cr(VI) removal performance. Environ. Sci. Technol..

[7] Kong X.K., Han Z.T., Zhang W., Le Song L., Li H. (2016). Synthesis of zeolite-supported microscale zero-valent iron for the removal of Cr6+ and Cd2+ from aqueous solution. J. Environ. Manag..

[8] Ifthikar J., Shahib I.I., Jiang W., Senthilnithy R., Elkhlifi Z., Wang J., Zhuqi Chen Z.Q. (2023). Review on technologies for the development of effective and practical chromate removal from wastewaters. J. Environ. Chem. Eng..

[9] Dong H., Deng J., Xie Y., Zhang C., Jiang Z., Cheng Y., Hou K., Zeng G. (2017). Stabilization of nanoscale zero-valent iron (nZVI) with modified biochar for Cr(VI) removal from aqueous solution. J. Hazard. Mater..

[10] Zhao R.R., Zhou Z.M., Zhao X.D., Jing G.H. (2019). Enhanced Cr(VI) removal from simulated electroplating rinse wastewater by amino-functionalized vermiculite-supported nanoscale zero-valent iron. Chemosphere.

[11] Chen Y.N., Liang W.Y., Li Y.P., Wu Y.X., Chen Y.R., Xiao W., Zhao L., Zhang J.C., Li H. (2019). Modification, application and reaction mechanisms of nano-sized iron sulfide particles for pollutant removal from soil and water: A review. Chem. Eng. J..

[12] Zhuang M., Wang H., Qi L., Cui L.Q., Quan G.X., Yan J.L. (2021). Production of activated biochar via a self-blowing strategy-supported sulfidated nanoscale zerovalent iron with enhanced reactivity and stability for Cr(VI) reduction. J. Clean. Prod..

[13] Cong Y.Q., Shen L.D., Wang B.M., Cao J.L., Pan Z.X., Wang Z.Y., Wang K., Li Q.B., Li X.C. (2022). Efficient removal of Cr(VI) at alkaline pHs by sulfite/iodide/UV: Mechanism and modeling. Water Res..

[14] Gao J., Yang L.Z., Liu Y.Y., Shao F.L., Liao Q.J.H., Shang J.G. (2018). Scavenging of Cr(VI) from aqueous solutions by sulfide modified nanoscale zero-valent iron supported by biochar. J. Taiwan Inst. Chem. Eng..

[15] Jia Z.Z., Shu Y.H., Huang R.L., Liu J.G., Liu L.L. (2018). Enhanced reactivity of nZVI embedded into supermacroporous cryogels for highly efficient Cr(VI) and total Cr removal from aqueous solution. Chemosphere.

[16] Ma Y.C., Jiang S.J., Zhong J., Chen X.K., Shu Y.H. (2022). Reactivity enhancement of ferrous sulfide by Poly-Sodium Acrylate cryogels on aqueous Cr(VI) removal: Performance and mechanism. J. Environ. Chem. Eng..

[17] Ifthikar J., Chen Z., Chen Z., Jawad A. (2020). A self-gating proton-coupled electron transfer reduction of hexavalent chromium by core-shell SBA-Dithiocarbamate chitosan composite. J. Hazard. Mater..

[18] Ifthikar J., Ibran Shahib I., Jawad A., Gendy E.A., Wang S., Wu B., Chen Z., Chen Z. (2021). The excursion covered for the elimination of chromate by exploring the coordination mechanisms between chromium species and various functional groups, Coord. Chem. Rev..

[19] Liu W., Jin L., Xu J., Liu J., Li Y., Zhou P., Wang C., Dahlgren R.A., Wang X. (2019). Insight into pH dependent Cr(VI) removal with magnetic Fe_3_S_4_. Chem. Eng. J..

[20] Luo H., Fu F.F.L., Tang B. (2023). Ferrous sulfide supported on modified diatomite for the removal of Cr(VI): Performance and mechanism. Colloids Surf. A Physicochem. Eng. Asp..

[21] Ajouyed O., Hurel C., Ammari M., Allal L.B., Marmier N. (2010). Sorption of Cr(VI) onto natural iron and aluminum (oxy)hydroxides: Effects of pH, ionic strength and initial concentration. J. Hazard. Mater..

[22] Loo S.L., Krantz W.B., Fane A.G., Gao Y.B., Lim T.T., Hu X. (2015). Bactericidal Mechanisms revealed for rapid water disinfection by super absorbent cryogels decorated with silver nanoparticles. Environ. Sci. Technol..

[23] Yuan Y., Wang L.P., Gao L.Z. (2020). Nano-Sized Iron Sulfifide: Structure, Synthesis, Properties, and Biomedical Applications. Front. Chem..

[24] Loo S.L., Fane A.G., Lim T.T., Krantz W.B., Liang Y.N., Liu X., Hu X. (2013). Super absorbent cryogels decorated with silver nanoparticles as a novel water technology for point-of-use disinfection. Environ. Sci. Technol..

[25] Wang W.H., Hu B.B., Wang C., Liang Z.J., Cui F.Y., Zhiwei Zhao Z.W., Yang C. (2020). Cr(VI) removal by micron-scale iron-carbon composite induced by ball milling: The role of activated carbon. Chem. Eng. J..

[26] Sapsford D., Barnes A., Dey M., Williams K., Jarvis A., Younger P., Liang L. Iron and manganese removal in a vertical flow reactor for passive treatment of mine water. Proceedings of the 7th International Conference on Acid Rock Drainage (ICARD).

[27] Wang W.H., Gao P., Yang C., Zhao Z.W., Zhen S.C., Zhou Y.X., Zhang T.T. (2022). Separable and reactivated magnetic mZVAl/nFe_3_O_4_ composite induced by ball milling for efficient adsorption-reduction-sequestration of aqueous Cr(VI). Sep. Purif. Technol..

[28] Lv X., Qin X., Wang K., Peng Y., Wang P., Jiang G. (2019). Nanoscale zero valent iron supported on MgAl-LDH-decorated reduced graphene oxide: Enhanced performance in Cr(VI) removal, mechanism and regeneration. J. Hazard. Mater..

[29] Varadharajan C., Belle H.R., Bill M., Brodie E.L., Conrad M.E., Han R.Y., Irwin C., Larsen J.T., Lim H.C., Molins S. (2017). Reoxidation of Chromium(III) products formed under different biogeochemical regimes. Environ. Sci. Technol..

[30] Jeong D., Kim K., Min D.W., Choi W.Y. (2015). Freezing-Enhanced Dissolution of Iron Oxides: Effects of Inorganic Acid Anions. Environ. Sci. Technol..

[31] Lv J.F., Tong X., Zheng Y.X., Xie X., Huang L.Y. (2018). Reduction of Cr(VI) with a relative high concentration using different kinds of zero-valent iron powders: Focusing on effect of carbon content and structure on reducibility. J. Cent. South Univ..

[32] Maleh H.K., Ayati A., Ghanbari S., Orooji Y., Tanhaei B., Karimi F., Alizadeh M., Rouhi J., Li Fu L., Sillanpää M. (2021). Recent advances in removal techniques of Cr(VI) toxic ion from aqueous solution: A comprehensive review. J. Mol. Liq..

[33] Zhao S., Chen Z., Wang B., Shen J., Zhang J., Li D. (2018). Cr(VI) removal using different reducing agents combined with fly ash leachate: A comparative study of their efficiency and potential mechanisms. Chemosphere.

[34] Zhang J., Chen L.P., Yin H.L., Jin S., Liu F., Chen H.H. (2017). Mechanism Study of humic acid functional groups for Cr(VI) retention: Two-dimensional FTIR and C-13 CP/MAS NMR correlation spectroscopic analysis. Environ. Pollut..

[35] Abdolali A., Ngo H.H., Guo W., Zhou J.L., Zhang J., Liang S., Chang S.W., Nguyen D.D., Liu Y. (2017). Application of a breakthrough biosorbent for removing heavy metals from synthetic and real wastewaters in a lab-scale continuous fixed-bed column. Bioresour. Technol..

[36] Pholosi A., Naidoo E.B., Ofomaja A.E. (2020). Batch and continuous flow studies of Cr(VI) adsorption from synthetic and real wastewater by magnetic pine cone composite. Chem. Eng. Res. Des..

[37] Gheju M., Iovi A. (2006). Kinetics of hexavalent chromium reduction by scrap iron. J. Hazard. Mater..

[38] Melitas N., Chuffe-Moscoso O., Farrell J. (2001). Kinetics of soluble chromium removal from contaminated water by zerovalent iron media: Corrosion inhibition and passive oxide effects. Environ. Sci. Technol..

[39] Lyu S.L., Liu T., Wang X., Zuo K.X., Xie Y.H. (2023). Removal and mechanism study of Cr(VI) in water by sludge biochar-supported nano-ferrous sulfide. China Environ. Sci..

[40] Lin C.L., Zhong L.Y., Zhong X.L., Wei B.Y., Yin J.Y. (2023). Adsorption-reduction reaction between bagasse-prepared biochar and Cr(VI). J. Agro-Environ. Sci..

[41] Liu S.Y., Han J.C., Ma D.Q., Liu H.X., Fang M., Tan X.L. (2023). MXene@MOF for synergetic removal of Cr(VI) by adsorption and reduction. Colloids Surf. A Physicochem. Eng. Asp..

[42] Chen Z.L., Zhang Y.N., Guo J.Z., Chen L., Li B. (2023). Enhanced removal of Cr(VI) by polyethyleneimine-modified bamboo hydrochar. Environ. Sci. Pollut. Res..

[43] Ren T.F., Zhang Y.X., Liu J.Q., Zhang Y.Q., Yang S.Y. (2020). Ethanol-assisted mechanical activation of zero-valent aluminum for fast and highly efficient removal of Cr(VI). Appl. Surf. Sci..

[44] Chen H.X., Dou J.F., Xu H.B. (2017). Removal of Cr(VI) ions by sewage sludge compost biomass from aqueous solutions: Reduction to Cr(III) and biosorption. Appl. Surf. Sci..

[45] Wang X., Liu W., Fu H.F., Yi X.H., Wang P., Zhao C., Wang C.C., Zheng W.W. (2019). Simultaneous Cr(VI) reduction and Cr(III) removal of bifunctional MOF/Titanate nanotube composites. Environ. Pollut..

