# Supplementary material for: nFeS Embedded into Cryogels for High-Efficiency Removal of Cr(VI): From Mechanism to for Treatment of Industrial Wastewater
Source: Gels. 2024 Jan 11;10(1):56. doi: 10.3390/gels10010056 (PMC10815210; doi:10.3390/gels10010056)
Supplement: Supplementary file 1 [file gels-10-00056-s001.zip › gels-2802468-supplementary.pdf]

nFeS Embedded into Cryogels for high efficiency removal of Cr (VI)：From mechanism to for Treatment of Industrial Wastewater

Peng Xu [a]，Shaojun Jiang [b*]

a. School of Environment, South China Normal University, Guangzhou 510006, China

b. Institute of Agricultural Resources and Environment, Guangdong Academy of Agricultural Sciences, Guangzhou 510640, China

* Corresponding author: Shaojun Jiang

E-mail address: shaojunj93@163.com (J S. Jiang).

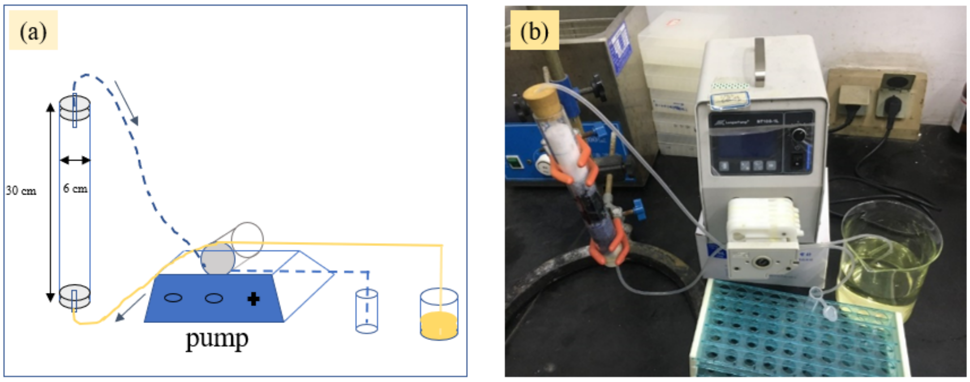

Fig. S1 Column experiments of reaction system.

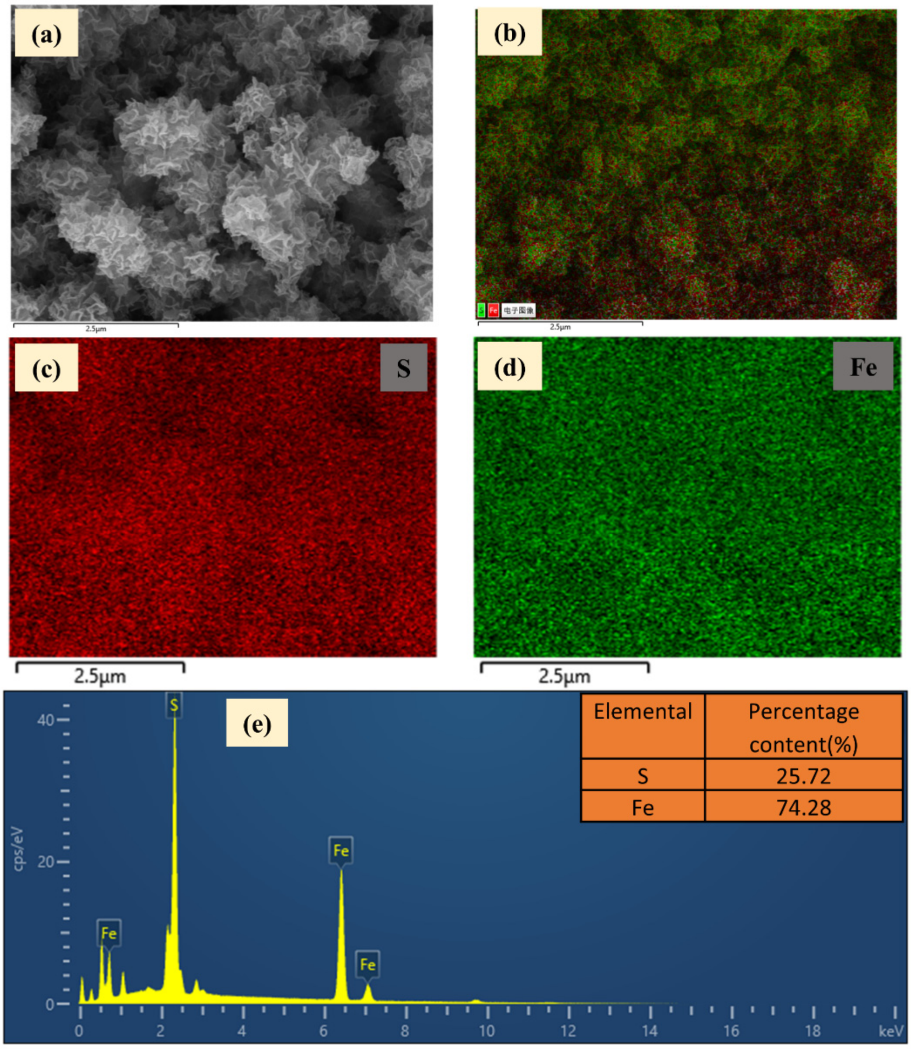

Fig.S2 SEM-EDS elemental mapping images of PSA-nFeS.

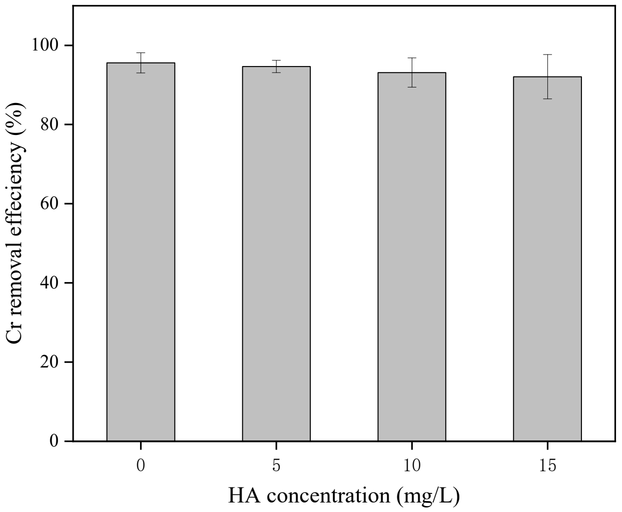

Fig. S3 Effect of Cr(VI) remove capacity of HA

Table S1. Physico-chemical parameters of industrial effluents.

Parameters	Tannery effluent	Water quality after reacted
pH	4.48	5.24
Turbidity, NTU	37	1.7
Total suspended solids	186.12	32
Sulphate	8208	1002
Chloride	864.02	164.02
Iron	5.75	1.32
BOD	1935	-
COD	9504	-
Total chromium (mg/L)	767	12
Chromium(VI) (mg/L)	52.45	0.5

Note: Dilute the mother liquor 10 times as reaction solution. Column experimental reaction parameters: Co (mg/L)=25, Filling doses (g nFeS)=0.056, Q (L/h)=0.54. “-” mean not measured.

Table S2 A comparison of treatment effect for Cr ions by different material

Material	Mechanisms	Q_m（mg/g）	References
PSA-nZVI	Reduction and	128.70	(Jia et al., 2018)

	adsorption		
PSA-nFeS	Reduction and adsorption	282.43	(Ma et al., 2022)
MBC	adsorption	21.01	(Lyu et al., 2023)
HC	adsorption	125.79	(Lin et al., 2023)
RC	adsorption	100.60	(Lin et al., 2023)
Polyethyleneimine-modified hydrochar (PEI-HC)	Reduction to Cr(III) and biosorption	528.41	(Chen et al.,2023)
Ethanol-assisted mechanical activation of zero-valent aluminum	Reduced and precipitated	5.23	(Ren et al., 2020)
Sewage sludge compost biomass	Reduction to Cr(III) and biosorption	Cr(Ⅵ) 1.87, Cr(Ⅲ) 34.60	(Chen et al.,2017)
MXene@MOF	Adsorption and reduction	224.46	(Liu et al., 2023)
Bifunctional MOF/Titanate nanotube composites	Photocatalysis reduction and adsorption	-	(Wang et al., 2019)

Qm: Maximum sorption capacity for Cr; MBC mean sludge biochar-supported nano-ferrous sulfide；Biochar（RC and HC）were prepared from bagasse by carbonization at 600 ℃ and 800 ℃；

References:

Lyu, S L., Liu, T., Wang, X., et al. Removal and mechanism study of Cr(VI) in water by sludge biochar-supported nano-ferrous sulfide . China Environmental Science, 2023,43(8):3935-3945.

Lin, C L., Zhong, L Y., Zhong, X L., et al. Adsorption-reduction reaction

between bagasse-prepared biochar and Cr（Ⅵ）. Journal of Agro-Environment Science, 2023, 42（10）：2335-2345.

Liu, S Y., Han, J C., Ma, D Q., Liu, H X., Fang, M., Tan, X L., MXene@MOF for synergetic removal of Cr(VI) by adsorption and reduction, Colloids and Surfaces A: Physicochemical and Engineering Aspects 678, 5,2023, 132438.

Chen. Z L., Zhang. Y N., Guo. J Z., Chen. L, Li, B., Enhanced removal of Cr(VI) by polyethyleneimine-modified bamboo hydrochar. Environmental Science and Pollution Research, 2023, 30, 94185–94194.

Ren, T. F., Zhang, Y X., Liu, J Q., Zhang, Y Q., Yang, S Y., Ethanol-assisted mechanical activation of zero-valent aluminum for fast and highly efficient removal of Cr(VI). Applied Surface Science. 2020, 533, 15 147543.

Chen, H X., Dou, J F., Xu, H B., (2017) Removal of Cr(VI) ions by sewage sludge compost biomass from aqueous solutions: Reduction to Cr(III) and biosorption. Applied Surface Science.2017, 425, 15, 728-735

Wang, X., Liu, W., Fu, H F., Yi, X H., Wang, P., Zhao, C., Wang, C C., Weiwei Zheng, W W.,Simultaneous Cr(VI) reduction and Cr(III) removal of bifunctional MOF/Titanate nanotube composites. Environmental Pollution 2019, 249, 502-511.
